# Second order topology in a band engineered Chern insulator

**DOI:** 10.1038/s41598-024-52321-y

**Published:** 2024-01-22

**Authors:** Srijata Lahiri, Saurabh Basu

**Affiliations:** Department of Physics, Indian Institute of Technology Guwahati, Guwahati, Assam 781039 India

**Keywords:** Materials science, Nanoscience and technology, Physics

## Abstract

Haldane model is a celebrated tight binding toy model of a Chern insulator in a 2D honeycomb lattice that exhibits quantized Hall conductance in the absence of an external magnetic field. In our work, we deform the bands of the Haldane model smoothly by varying one of its three nearest neighbour hopping amplitudes ($t_1$), while keeping the other two (*t*) fixed. This breaks the $C_3$ symmetry of the Hamiltonian, while the $M_x*T$ symmetry is preserved. The symmetry breaking causes the Dirac cones to shift from the **K** and the **K**$'$ points in the Brillouin zone (BZ) to an intermediate **M** point. This is evident from the Berry curvature plots which show a similar shift in the corresponding values as a function of the deformation parameter, namely $\frac{t_1}{t}$. We observe two different topological phases of which, one is a topological insulator (TI) and the other is a second order topological insulator (SOTI). The Chern number (*C*) remains perfectly quantized at a value of $C=1$ for the TI phase and it goes to zero in the SOTI phase. Furthermore, the evolution of the Wannier charge center (WCC) as the band is smoothly deformed shows a jump in the TI phase indicating the presence of conducting edge modes. We also study the SOTI phase and diagonalize the real space Hamiltonian on a rhombic supercell which shows the presence of in-gap zero energy corner modes. The polarization of the system, namely $p_x$ and $p_y$, are evaluated, along the *x* and the *y* directions, respectively. We see that both $p_x$ and $p_y$ are quantized in the SOTI phase owing to the presence of the inversion symmetry of the system. Finally we establish the SOTI phase as an example of a topological phase with zero Berry curvature and provide an analogy with the two dimensional Su–Schrieffer–Heeger model.

## Introduction

The field of topologicals insulators (TI) deals with systems that have a gapped bulk in *d* dimensions but show topological states in the $d-1$ dimensional boundary^1–4^. Higher order topological insulators (HOTI) are an extension to topological insulators, where robust non-trivial boundary states are found in dimensions less than $d-1$ for a bulk that is *d* dimensional^5–22^. For instance, second order TIs exhibit the signature of non-trivial topology in $d-2$ dimensions. This gives rise to corner modes for a two-dimensional and hinge modes for a three-dimensional bulk. The conventional definition of bulk-boundary correspondence fails in higher order topology. What we rather find here is a refined bulk-boundary correspondence. Higher order topology has been discussed in the context of a spectrum of systems called topological crystalline insulators which are topological systems protected by crystalline symmetries namely inversion, rotation etc.^23^. The refined bulk boundary correspondence has also been extended to higher order electric multipole insulators where the systems feature localized corner excitations as a result of sharply quantized higher order multipole moments^24,25^. Prospective material candidates like strained SnTe and surface modified BiSe and BiTe which have theoretically proven to show a distinct higher order topological phase, have also been studied in detail^26^. Several higher order topological insulators have previously been considered as trivial insulators due to the absence of boundary states. A significant example of this scenario being Bismuth which was considered to be a trivial insulator, but rather shows higher order hinge modes owing to double band inversion in the bulk^12^. In this work, we discuss a similar scenario that shows higher order topology.

**Figure 1:**
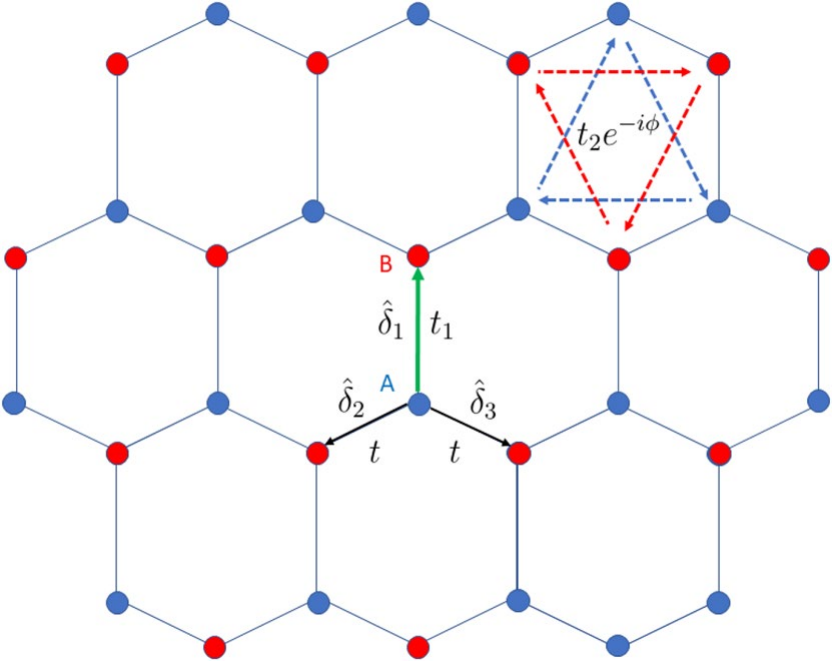
Schematic diagram of the honeycomb lattice. The two sublattices are denoted as *A* and *B*. The nearest neighbour vector directions are shown as $\hat{\delta }_1$, $\hat{\delta }_2$ and $\hat{\delta }_3$. The hopping along the direction $\hat{\delta }_1$ is given by $t_1$ (shown in green), while it is given by *t* in the other two directions (shown in black). The second neighbour hoppings are given by dashed lines and have an amplitude $t_2$.

The Haldane model or Chern insulator is a well known model of a two-dimensional topological insulator built on a hexagonal lattice which exhibits the presence of robust chiral modes at the edges of the system^27^. It was introduced with the motivation to realise non-zero quantized Hall conductance in a honeycomb lattice in the absence of an external magnetic field. The main aim was to gap out the Dirac cones in the bulk Hamiltonian of Graphene by breaking time reversal symmetry via complex next nearest neighbour hopping amplitudes. This opens up non-trivial gaps at the **K** and **K**$'$ points in the Brillouin zone of the system when the second neighbour hopping (say $t_2$) satisfies the condition $|t_2|\ge \frac{m}{3\sqrt{3}\sin \phi }$. Here *m* is the Semenoff mass and $\phi$ denotes the phase of the complex hopping amplitude. The system exhibits quantized Hall conductance, which in turn is a result of integer values of the topological invariant, namely the Chern number. The Chern number is a signature of quantized charge transport across the bulk of a system due to adiabatic transformation of the Hamiltonian along a closed loop in the parameter space^28^. This, in turn can be obtained by integrating the Berry phase over the same closed loop. Previous studies on the Haldane model reported a transition from a topological to a trivial phase under a smooth deformation of its bandstructure which is brought about by tuning the amplitude of one of the nearest neighbour (NN) hopping parameter (say $t_1$) while keeping other two NN parameters (say *t*) fixed^29^. This deformation breaks the $C_3$ symmetry of the hexagonal lattice. In our work, however, we show that this takes the system from a TI to an HOTI phase, or more specifically a second order topological insulator phase (SOTI). States localised at the corners of a rhombic supercell of the hexagonal lattice have been found when $\frac{t_1}{|t|}$ exceeds a certain critical value. These localized states in dimensions $d-2$ are symbolic of a higher order topological phase. We evaluate the Chern numbers corresponding to both the TI and the SOTI phase. The Chern number exhibited in the SOTI phase is zero, owing to the absence of conducting modes at the edges of the system. Further, we also study the evolution of the Wannier charge center along a closed one dimensional loop in the Brillouin zone both for the TI and the SOTI phase. The Wannier charge center which represents the average position of charge in the unit cell of a crystal lattice bears the same information as is carried by surface energy bands^30,31^. Furthermore, to characterize our SOTI phase we calculate the polarization along the *x* and *y* directions which are bulk topological invariants obtained as the integral of the Berry connection^10,32^. Interestingly, we show here that the second neighbour hopping $t_2$ which breaks time reversal symmetry, and is of prime importance in the TI phase, holds no significance in the SOTI phase. This is in contrast to the work done in Ref.^32^, where both the nearest neighbour and next nearest neighbour hopping amplitudes are modified. We show that the insignificance of $t_2$ in the SOTI phase is of prime importance in establishing that the SOTI phase is an example of a topological phase with zero Berry curvature. A study on the topological phases of strained pristine Graphene, has also been done in Ref.^33^.

**Figure 2:**
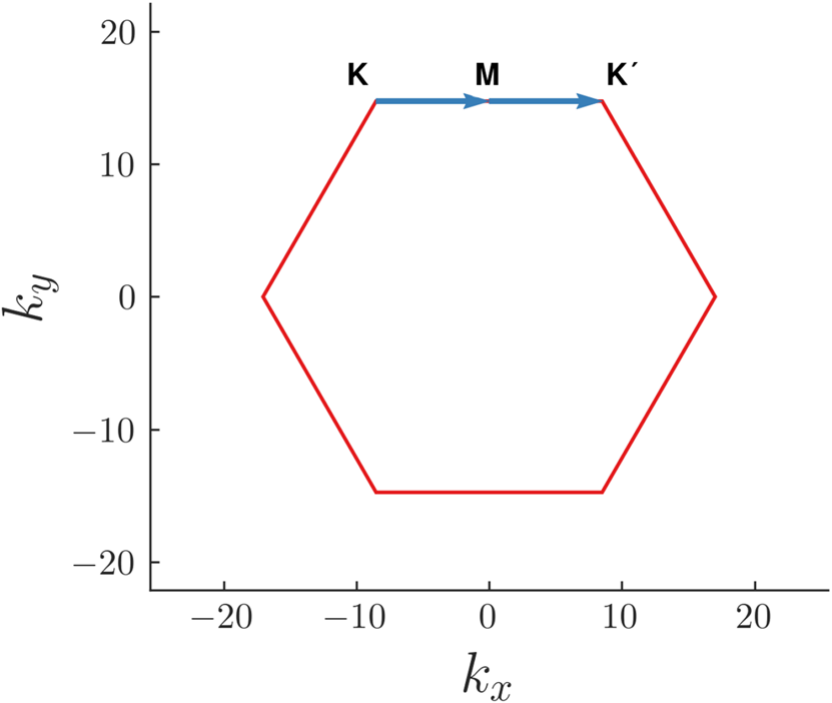
Brillouin zone for the honeycomb lattice with the path (**K**$\rightarrow$**M**$\rightarrow$**K**$'$) shown along which the bandstructure is calculated.

The paper is structured as follows. In “The Hamiltonian”, we discuss the tight binding Hamiltonian for the Haldane model and discuss the deformation of the bandstructure as a function of ratio of the two NN hopping amplitudes ($\frac{t_1}{t}$). We define the Chern number as a topological invariant for the TI phase and show that it goes to zero as the system transcends to a higher order topological insulator. We also calculate the Wannier charge center of the system which is equivalent to calculating the Berry phase along one particular direction (say $k_x$) of the 2D BZ and study its evolution over the momentum in the other direction ($k_y$) for both the TI and the SOTI phases. In “Second order topological phase”, we plot real space eigenvalue spectra of a rhombic supercell of the Haldane model in the SOTI phase and show the presence of zero energy modes that are distinctly separate from the bulk. Further, the probability density of these states are shown which confirm that they are localized at the two corners of the rhombic supercell. We define bulk polarization as a topological invariant for the SOTI phase, and show that it is quantized owing to the inversion symmetry of the system. Finally in “Analysis of the second order topological phase”, we analyse the SOTI phase and establish an analogy with the two dimensional Su–Scrieffer–Heeger (SSH) model. We also establish that the second neighbour hopping amplitude $t_2$ plays no role in the higher order topology of the deformed Haldane model and hence it can be considered as a classic example of a topological phase with zero Berry curvature.

## The Hamiltonian

**Figure 3:**
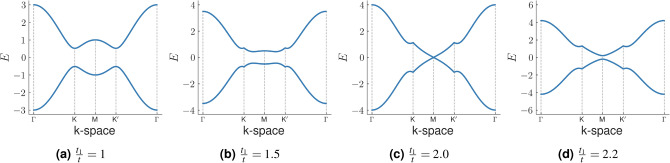
The bandstructure of the deformed Haldane model is plotted as a function of $\xi =\frac{t_1}{t}$ along the $\Gamma \rightarrow$**K**$\rightarrow$**M**$\rightarrow$**K**$'$ $\rightarrow \Gamma$ path in the BZ of the honeycomb lattice for (**a**) $\xi =1$, (**b**) $\xi =1.5$, (**c**) $\xi =2.0$, (**d**) $\xi =2.2$. We observe that with increasing $\xi$ the band minima points slowly shift towards the **M** point of the BZ from the **K** and **K**$'$ points. At $\xi =2$ the band gap vanishes at the **M** point indicating a possible topological phase transition. Beyond this point, at $\xi >2$, the band gap reopens. The parameters *t*, $t_2$, $\phi$ and *m* have been fixed at 1, 0.1, $\frac{\pi }{2}$ and 0.

**Figure 4:**
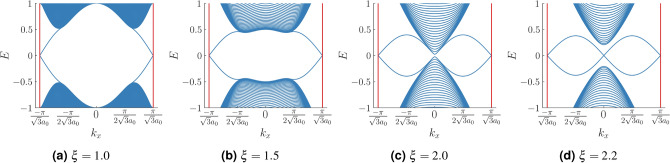
The bandstructure for a zig-zag ribbon-like configuration of the Haldane model is shown. For $\xi <2$, in (**a**,**b**), we see edge modes crossing the bulk gap. The system is in the TI phase in this region. (**c**) At $\xi =2$, the bandstructure undergoes a gap closing transition. The closure is not perfect due to the limited number of lattice sites taken along ${\hat{a}}_2$. (**d**) Beyond $\xi =2$ we still see the presence of in-gap modes. However they do not traverse the bulk gap and can be easily removed by an adiabatic deformation of the Hamiltonian. We have kept the values of the parameters *t*, $t_2$, $\phi$ and *m* fixed at 1, 0.1, $\frac{\pi }{2}$ and 0. The number of unit cells along ${\hat{a}}_2$ (see text) is 127.

The Haldane model is defined on a honeycomb lattice as represented in Fig. 1. The vectors connecting the nearest neighbours are given by $\vec \delta _1=a_0(0,1)$, $\vec \delta _2=a_0(-\frac{\sqrt{3}}{2},-\frac{1}{2})$, $\vec \delta _3=a_0(\frac{\sqrt{3}}{2},-\frac{1}{2})$ where $a_0$ is the length of the nearest neighbour distance. The lattice vectors are given by $\vec {a}_1=\vec {\delta }_1-\vec {\delta }_2$ and $\vec {a}_2=\vec {\delta }_1-\vec {\delta }_3$. In our model, the NN hopping along the direction $\hat{\delta }_1$ is assumed to be $t_1$, while it is given by *t* in the directions $\hat{\delta }_2$ and $\hat{\delta }_3$. The time reversal symmetry of the system is broken by the next nearest neighbour (NNN) hopping which has a form $t_2e^{i\phi }$ where $\phi$ is the phase associated with the hopping. $\phi$ is positive (negative) for hopping along the anticlockwise (clockwise) direction. For our purpose, we keep $\phi$ fixed at $\frac{\pi }{2}$. The hexagonal lattice has two sublattices denoted by *A* and *B* in the figure. The Semenoff mass *m* is positive(negative) for the sublattice A(B). The NNN vectors, that is, the vectors connecting the nearest neighbour A-A (or B-B) atoms are given by, $\vec \nu _1=\vec \delta _3-\vec \delta _1$, $\vec \nu _2=\vec \delta _2-\vec \delta _3$ and $\vec \nu _3=\vec \delta _1-\vec \delta _2$. The real space Hamiltonian for the Haldane model can be written as,

1 $$\begin{aligned} \begin{aligned} H=\sum _{\langle i,j \rangle }t_{ij}c_i^\dagger c_j + \sum _{\langle \langle i,j \rangle \rangle }t_2e^{i\phi _{ij}}c_i^\dagger c_j + \sum _{i}m_ic_i^\dagger c_i + h.c. \end{aligned} \end{aligned}$$

where $c_i$ ($c_i^\dagger$) represent annihilation (creation) operators at lattice site *i*. We Fourier transform this Hamiltonian to obtain the tight binding Hamiltonian for the deformed Haldane model,

2 $$\begin{aligned} \begin{aligned} H(k)&=\Big [t_1\cos \vec {k}\cdot \vec {\delta _1} + \sum _{i=2,3}t\cos {\vec {k}\cdot \vec {\delta _i}}\Big ]\sigma _x + \Big [t_1\sin \vec {k}\cdot \vec {\delta _1} + \sum _{i=2,3}t\sin {\vec {k}\cdot \vec {\delta _i}}\Big ]\sigma _y + 2t_2\Big [\sin \vec k\cdot (\vec {\delta _3}-\vec {\delta _1})+\sin \vec k\cdot (\vec {\delta _2}-\vec {\delta _3})\\&+\sin \vec k\cdot (\vec {\delta _1}-\vec {\delta _2})\Big ]\sigma _z+m\sigma _z \end{aligned} \end{aligned}$$

**Figure 5:**
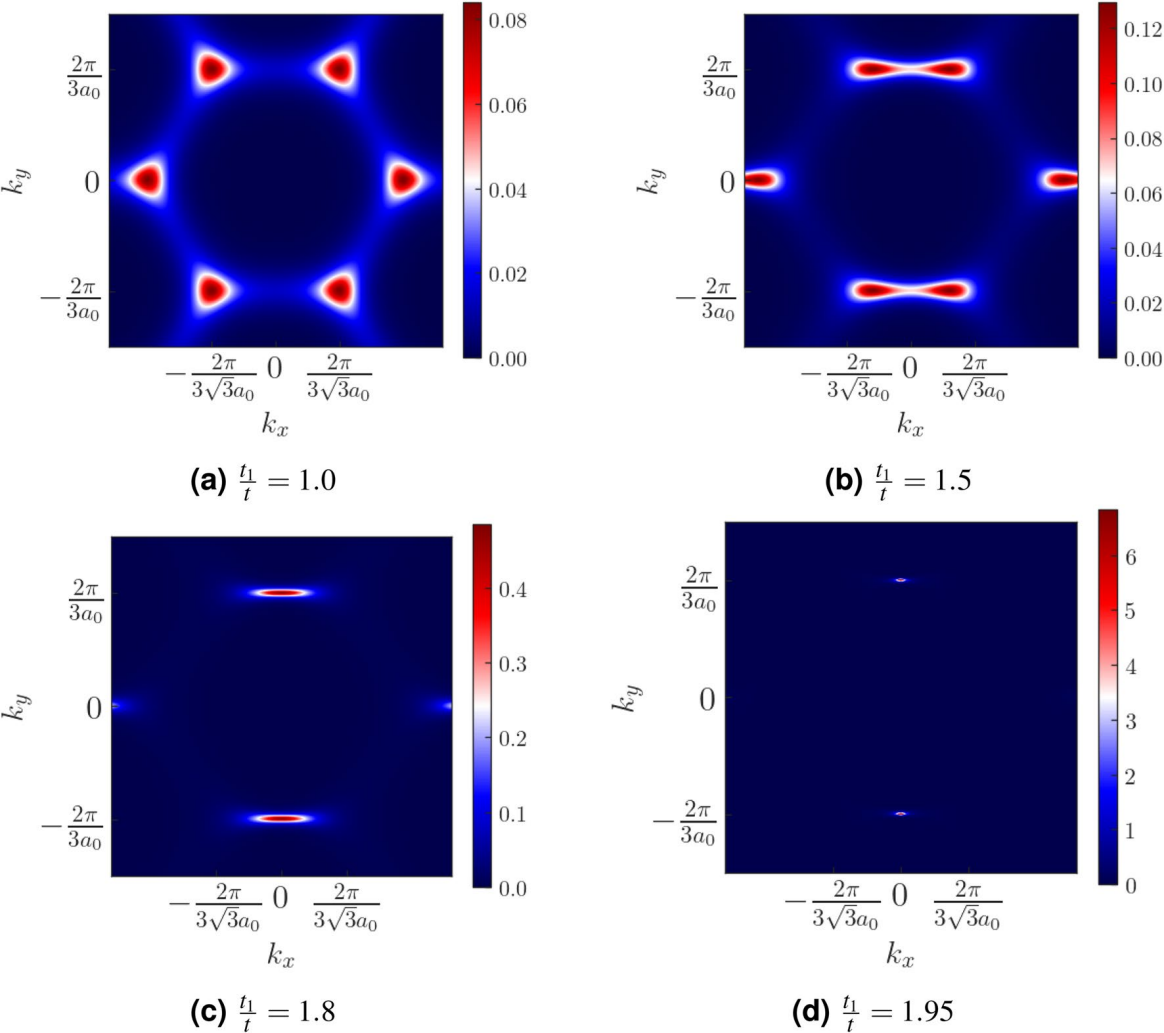
The Berry curvature for different values of $\xi =\frac{t_1}{t}$ are shown over the Brillouin zone of honeycomb lattice. For (**a**) $\xi =1$, the Berry curvature is confined near the **K** and **K**$'$ points of the BZ. With increasing values of $\xi$ in (**b**–**d**) the region of high concentrations shift towards the **M** point. This indicates a shift in the point of minimum band gap with increasing $\xi$. The values of the parameters *t*, $t_2$, $\phi$ and *m* are fixed at 1, 0.1, $\frac{\pi }{2}$ and 0.

Here $\sigma _x$, $\sigma _y$ and $\sigma _z$ denote the Pauli matrices. The bond in the direction $\hat{\delta }_1$ is referred to as the strong bond, while the bond in the other two directions are weak when compared with the former. The Hamiltonian here, has been written in the sublattice basis ($|c_{k_A}\rangle$, $|c_{k_B}\rangle$). It is to be noted that in the original Haldane model, the hopping is uniform along all three nearest neighbour vectors, that is, $t_1=t$. Furthermore, the deformed Haldane model breaks the $C_3$ symmetry which was otherwise present in the original counterpart. It however preserves a product of $M_x$ and *T*, where $M_x$ represents mirror symmetry about $x=0$ and *T* is the time reversal symmetry operator. It is to be noted that the action of the $M_xT$ symmetry on a Hamiltonian $H(\vec {k})$ is given by

3 $$\begin{aligned} (M_xT)H(\vec {k})(M_xT)^{-1} = H\{(M_xT)\vec {k}\}=H\{M_x(-k_x, -k_y)\}=H(k_x, -k_y) \end{aligned}$$

For the Haldane Hamiltonian, the $M_xT$ symmetry is denoted by $\sigma _0K$, where *K* represents complex conjugation and $\sigma _0$ represents identity. The action of the operator $\sigma _0$ is understood as follows. For an infinite lattice, the action of $M_x$ does not change the sublattice structure and resembles identity. The entire operation of $M_xT$ hence boils down to complex conjugation. Physically, the $M_x$ operator changes the direction of the second neighbour hopping. The amplitude of the second neighbour hopping being direction dependent, changes the physical structure of the Hamiltonian and causes the clockwise hopping to carry a positive phase. However, the time reversal symmetry changes the sign of the phase and re-establishes the previous structure of the Hamiltonian.

We first discuss the bandstructure of the deformed Haldane model, as a function of the quantity $\xi =\frac{t_1}{t}$. This system shows two Dirac nodes at the **K** ($\frac{-2\pi }{3\sqrt{3} a_0}, \frac{2\pi }{3a_0}$) and **K**$'$ ($\frac{2\pi }{3\sqrt{3} a_0},\frac{2\pi }{3a_0}$) points in the BZ at $\xi =1$ when both $t_2$ and *m* are zero. The spectral gaps open up at these points when $t_2$ is rendered finite. For our purpose, we keep the value of $t_2$ fixed at 0.1*t* and the phase $\phi$ at $\frac{\pi }{2}$. The Semenoff mass *m* is also fixed at zero. To aid in our understanding, the BZ of the honeycomb lattice and the path in reciprocal space that has been used for the construction of the bandstructure have been shown in Fig. 2. As the value of $\xi$ is increased from a value 1, the gaps in the bandstructure diminish and shift away from the **K** and **K**$'$ points, as shown in Fig. 3. As $t_1$ becomes equal to 2*t* ($\xi$=2), the gap closes at the **M** point $(0, \frac{2\pi }{3a_0})$ in the BZ even when the time reversal symmetry remains broken. On increasing $\xi$ further, the gap reopens. As reported in previous studies, this gap closing was considered to induce a transition of the system from a TI to a trivial phase^34^. We however show that beyond $\xi =2$, the system enters into a SOTI phase.

Next, we study the bandstructure of a zig-zag semi-infinite ribbon-like configuration of the model with periodic boundary condition (PBC) along direction ${\hat{a}}_1$ and open boundary condition (OBC) along the direction ${\hat{a}}_2$, to show the presence of edge modes connecting the conduction and valence bands in the TI phase (Fig. 4). The width of the ribbon along the *y* direction is given by $a_0(\frac{3N}{2}+1)$, where *N* denotes the number of unit cells in the *y* direction. We have chosen *N* to be equal to 127. It can be observed that for $\xi <2$, the system shows the presence of modes that traverse the bulk energy gap (Fig. 4a,b). Exactly at $\xi =2$ (Fig. 4c), the gap closes (the tiny gap seen in the figure at $k_x=0$ will vanish for large longitudinal system dimensions). For $\xi >2$, the bandstructure still shows the presence of in gap modes. However they are completely detached from the bulk and carry no topological significance (Fig. 4d). These modes correspond to counterpropagating edge states that cancel each other at each boundary of the ribbon and gives rise to a trivial insulating phase.

To characterize this topological insulator phase, we evaluate the Chern number which is a topological invariant obtained by integrating the Berry connection over a closed loop in the BZ. It is directly related to the conductivity of the edge modes. To calculate the Chern number, we first calculate the Berry curvature of the system. The Berry curvature is given as^35,36^,

4 $$\begin{aligned} \begin{aligned} \Omega (\vec k)&= \varvec{\nabla \times }{\vec {A}(\vec {k})}=i\biggl [ \biggl<\frac{\partial \psi (\vec k)}{\partial k_x}\biggl |\frac{\partial \psi (\vec k)}{\partial k_y}\biggl> - \biggl <\frac{\partial \psi (\vec k)}{\partial k_y}\biggl |\frac{\partial \psi (\vec k)}{\partial k_x}\biggl >\biggl ] \end{aligned} \end{aligned}$$

The Chern number is now calculated using,

5 $$\begin{aligned} \begin{aligned} C=\frac{1}{2\pi }\iint _{BZ}\Omega (\vec k)\cdot d\vec {S} \end{aligned} \end{aligned}$$

**Figure 6:**
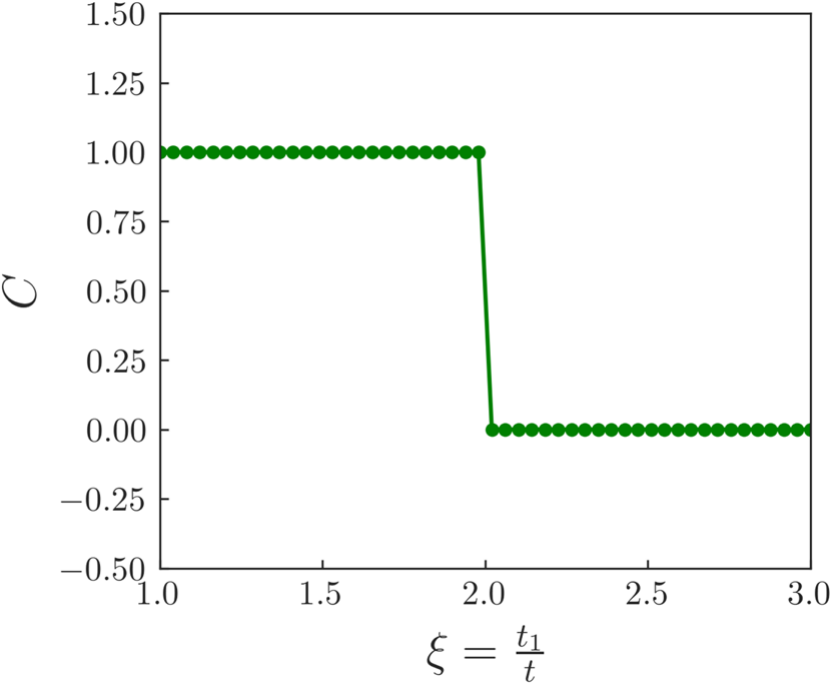
The Chern number is perfectly quantized at 1 for $\xi <2$, which is the TI phase. For $\xi >2$, the Chern number goes to 0. This indicates vanishing of the edge modes in the system which is evident from the bandstructure plots (Fig. 4) as well. The parameters *t*, $t_2$, $\phi$ and *m* are fixed at 1, 0.1, $\frac{\pi }{2}$ and 0, respectively.

We first plot the Berry curvature of the system over the entire BZ for different values of $\xi$ (Fig. 5). A non-zero Berry curvature is a direct consequence of the presence of Dirac nodes in the system. Our plot shows that at $\xi =1$, the Berry curvature is concentrated around the **K** and **K**$'$ points in the BZ (Fig. 5a). As $\xi$ is increased, the Berry curvature slowly shifts towards the *M* point (Fig. 5b–d). This is because with the increase in $\xi$, the $C_3$ symmetry, which kept the Dirac nodes pinned to the **K** and **K**$'$ points is broken. We integrate this Berry curvature over the BZ to obtain the Chern number *C*. We plot *C* as a function of $\xi$, as shown in Fig. 6. It can be clearly seen that *C* is perfectly quantized at 1 when $\xi <2$. Furthermore, for $\xi >2$, *C* goes to 0.

**Figure 7:**
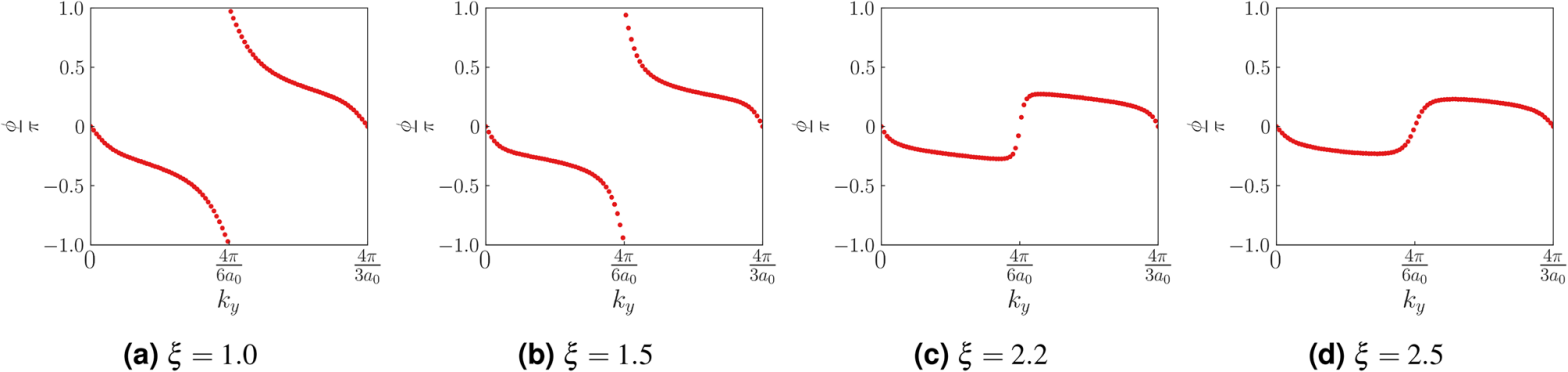
Wannier charge center (WCC) along the *x*-direction has been plotted as a function of momentum along the *y*-direction, namely $k_y$. (**a**,**b**) TI phase, where a non-trivial winding of the WCC, is noted as $k_y$ traverses a closed loop in the BZ. This is equivalent to having a non-trivial Chern number and hence a non-trivial bulk. The jump in WCC indicates a shift in the average position of the electrons in a unit cell. (**c**,**d**) The system here is no longer in the TI phase. The WCC trivially oscillates about zero as a function of $k_y$. We have taken *t*, $t_2$ and $\phi$ as 1, 0.1 and $\frac{\pi }{2}$, respectively. The Semenoff mass *m* is taken to be close to zero. Specifically we have taken $m=0.00001$.

Now, we look at the evolution of the Wannier charge center (WCC) over a closed loop in the BZ (Fig. 7) and study its behaviour as a function of $\xi$. For a 2D system, this is equivalent to calculating the Berry phase along one direction (say $k_x$) and evolving it as a function of momentum in the other direction (say $k_y$)^30,37^. This plot bears information about the bulk topology. In our case, we calculate the WCC along the *x* direction and look for its evolution as a function of $k_y$. The mathematical expression for calculating the WCC (or equivalently the Berry phase) is given as^38^,

6 $$\begin{aligned} \begin{aligned} \phi _n(k_y)=\int _{0}^{2\pi }A_n(k_x,k_y)dk_x \end{aligned} \end{aligned}$$

Here $\vec {A}_n(k_x, k_y)=-i\langle u_{nk}|\nabla _k|u_{nk}\rangle$, where *n* is the band index. In the TI phase, we see the WCC winding non-trivially as $k_y$ in increased from 0 to $2\pi$ (Fig. 7a,b). This indicates a shift in the charge center of the reference unit cell over a closed loop in $k_y$. Charge conservation in this case demands the presence of edge states that traverse the bulk energy gap and transfers an electron from the valence to the conduction band. We also plot the behaviour of the WCC in the region $\xi >2$. It can be clearly seen that the WCC shows no winding here and oscillates about zero. This hints at a topologically trivial phase.

## Second order topological phase

**Figure 8:**
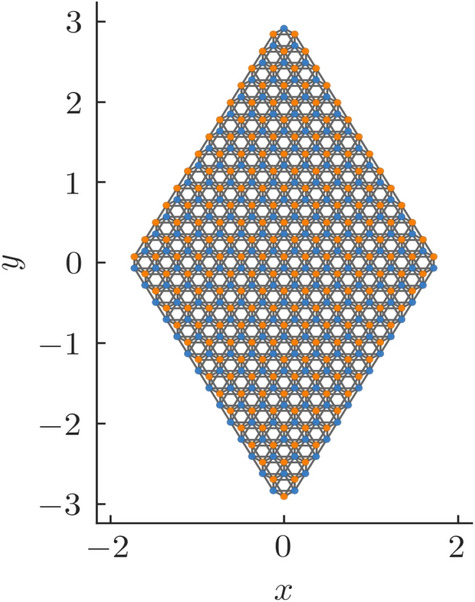
A rhombic supercell constructed using a honeycomb lattice which is used in our calculation for the SOTI phase.

**Figure 9:**
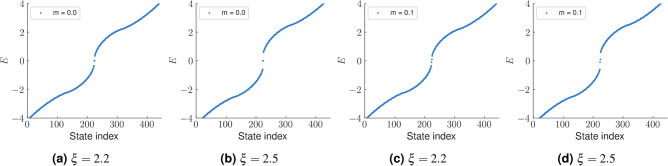
Real space Hamiltonian of the band deformed Haldane model has been diagonalized on the rhombic supercell. The resulting eigenvalues for (**a**) $\xi =2.2$ and (**b**) $\xi =2.5$ have been shown when the Semeneoff mass, $m=0$. The plots show the presence of zero energy eigenvalues which are distinctly separated from the bulk. It is to be noted that the system is no more in the TI phase for both the plots. In (**c**,**d**), the modes that were initially at zero energy, shift in the presence of a non-zero Semenoff mass. Again, the parameters *t*, $t_2$, and $\phi$ are fixed at 1, 0.1 and $\frac{\pi }{2}$, respectively.

**Figure 10:**
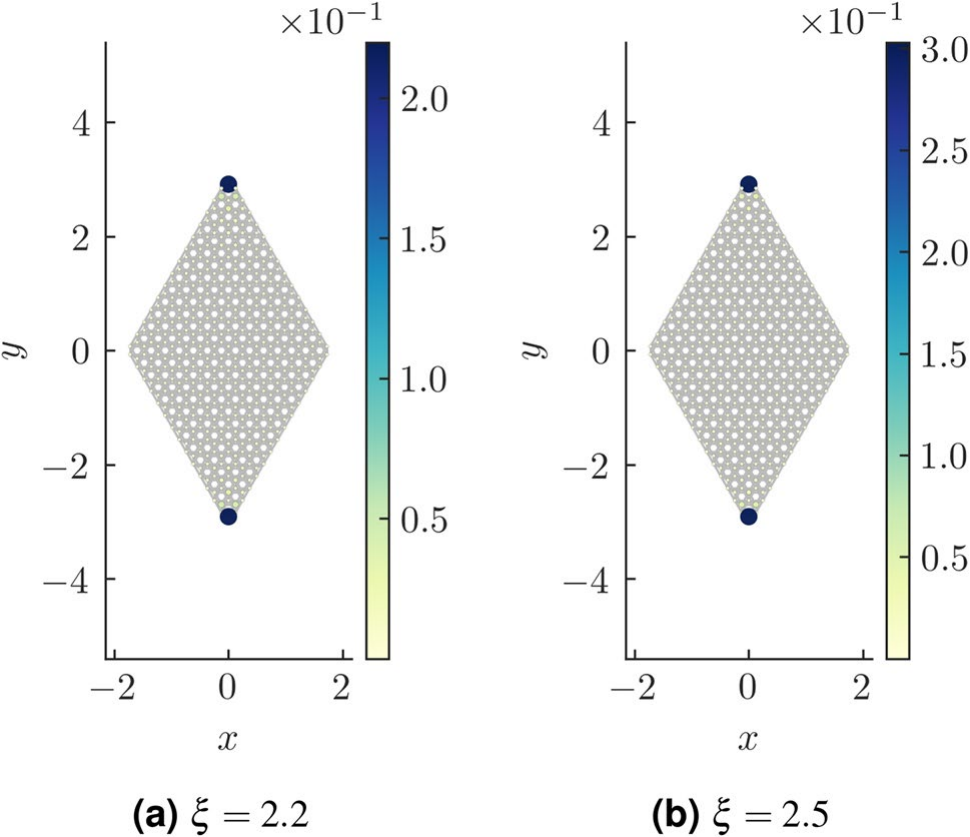
The probability density plots for the states at zero energy corresponding to the parameter values (**a**) $\xi =2.2$ and (**b**) $\xi =2.5$. The system is in the SOTI phase and demonstrates the presence of corner modes shown by deep blue colour. The other relevant parameters are kept same as mentioned in Fig. 9. The Semenoff mass is kept 0.

**Figure 11:**
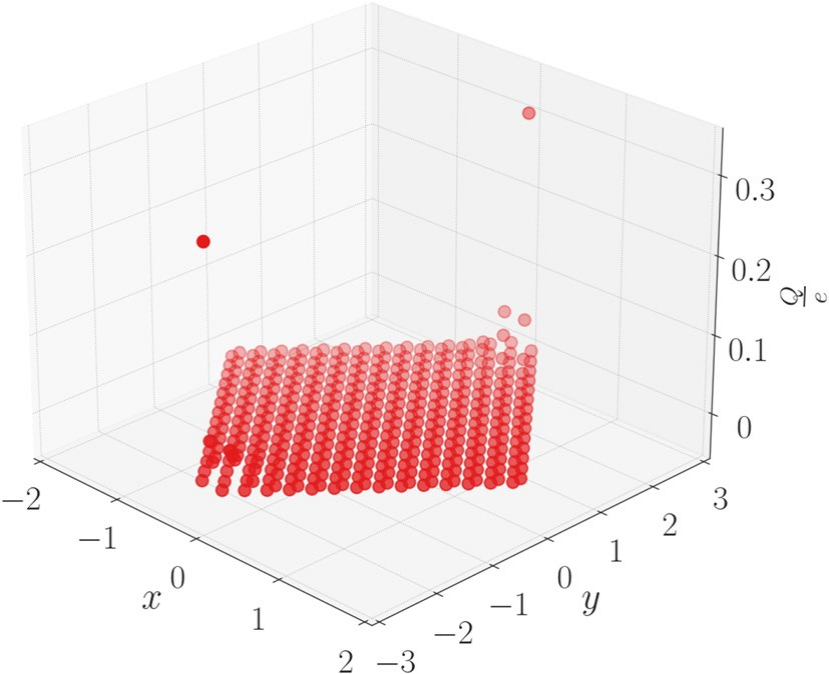
Fractional charge accumulation of 0.5 at the corners of the system for $\xi =2.5$ is shown. The accumulation is more noticeable for higher values of $\xi$.

We now focus on the region where $\xi >2$. The Chern number and the WCC both hint at this region being topologically trivial. We, however take a suitably formed rhombic supercell (Fig. 8) and diagonalize the real space Hamiltonian for this structure. The energy spectra of the real space Hamiltonian, for $\xi <2$ does not show any specific feature with regard to higher order topology (or more specifically, second order topology). However beyond the TI phase ($\xi \ge 2$), we see the presence of modes pinned at zero energy and distinctly separated from the bulk (Fig. 9). We further plot the probability density of these states which are found to be localized at two corners of the rhombus (Fig. 10). We had kept the value of the Semenoff mass *m* fixed at 0 which keeps the inversion symmetry in the system intact. On increasing the Semenoff mass, inversion symmetry in the system gets broken. The degeneracy of the states is lifted and the states drift from zero energy. They are, however no longer cleanly separated from the bulk. We also plot the fractional charge accumulation of the system at half filling (Fig. 11). It can be seen that the corners host a fractional charge density which tends to 0.5 on increasing $\xi$. The charge accumulation is also more precise for larger system sizes. The scenario corresponding to an armchair edge rectangular supercell has also been considered by us. The supercell has alternating zig-zag and armchair edges with the localization occurring at sites that are connected to the bulk of the system through weak bonds. We find a series of states around zero energy which are well separated from the bulk and localized at the “weak” sites along the zig-zag edges. However, this does not denote a conventional HOTI phase and hence we skipped further discussion on it.

To characterize the corner states of the rhombic supercell, we calculate the bulk polarization which is equivalent to the position of charge in a unit cell. In a crystal lattice it is difficult to unambiguously define a well defined unit cell that hosts a periodic distribution of charge. Such a definition is however important for the evaluation of polarization under the Clausius–Mosotti picture^39^. Hence, in bulk solids, we rely on the Wannier functions for the calculation of bulk polarization. Wannier functions are localized wave functions formed by suitable superposition of the delocalized Bloch wave functions. The expectation value of the position operator with respect to these Wannier functions is known as the Wannier charge center and is used to define polarization for a crystalline system. WCC, and hence the polarization in a direction $\alpha$ is given as^10,40^

7 $$\begin{aligned} \begin{aligned} p_\alpha ={{\bar{r}}_\alpha } = \langle w_n|r_\alpha |w_n\rangle =\frac{i}{S}\int _{BZ} d^{d}k \langle u_{nk}|\frac{\partial }{\partial k_{r_\alpha }}|u_{nk}\rangle \end{aligned} \end{aligned}$$

**Figure 12:**
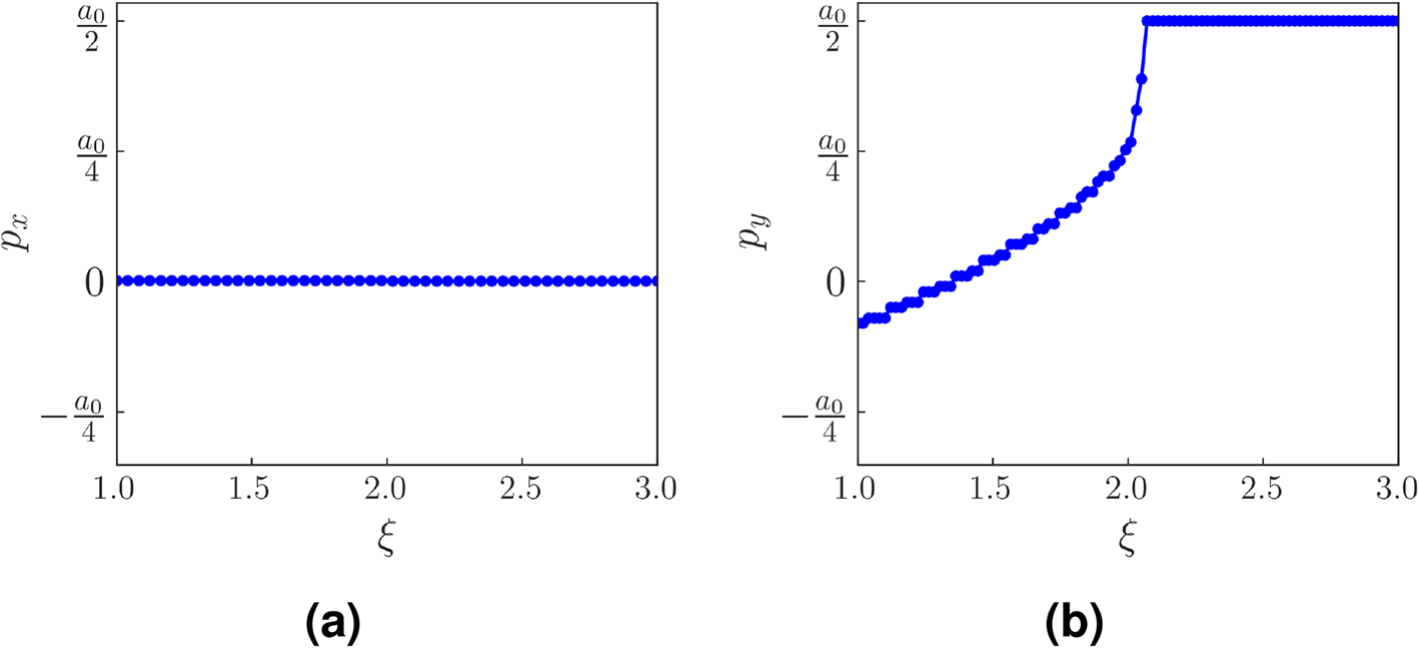
The polarization, $p_x$, $p_y$ as a function of $\xi$ has been shown. (**a**) $p_x$ vanishes uniformly both in the TI and SOTI phase. (**b**) $p_y$ is quantized in the SOTI phase at $\frac{a_0}{2}$ which is the center of the strong bond. The value of the Semenoff mass is kept infinitesimal (specifically $m=0.00001$). Other parameters are fixed as previously mentioned.

**Figure 13:**
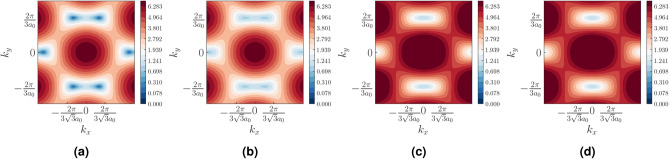
Band gap calculated for the fermionic dispersion in the TI phase and the SOTI phase are shown. (**a**) Here $\xi =1.5$ and $t_2=0$. This shows that there are Dirac nodes at the **K** and **K**$'$ points in the BZ. (**b**) Here $\xi =1.5$ and $t_2=0.1$. Clearly the gap has now opened. In (**c**,**d**) the system is in the SOTI phase. Here $\xi =2.5$ and $t_2=0$ and 0.1, respectively. Clearly, no topological phase transition occurs. Thus the two Hamiltonians corresponding to these parameters can be adiabatically connected. Here *t*, $\phi$ and *m* are fixed at 1, $\frac{\pi }{2}$ and 0, respectively.

**Figure 14:**
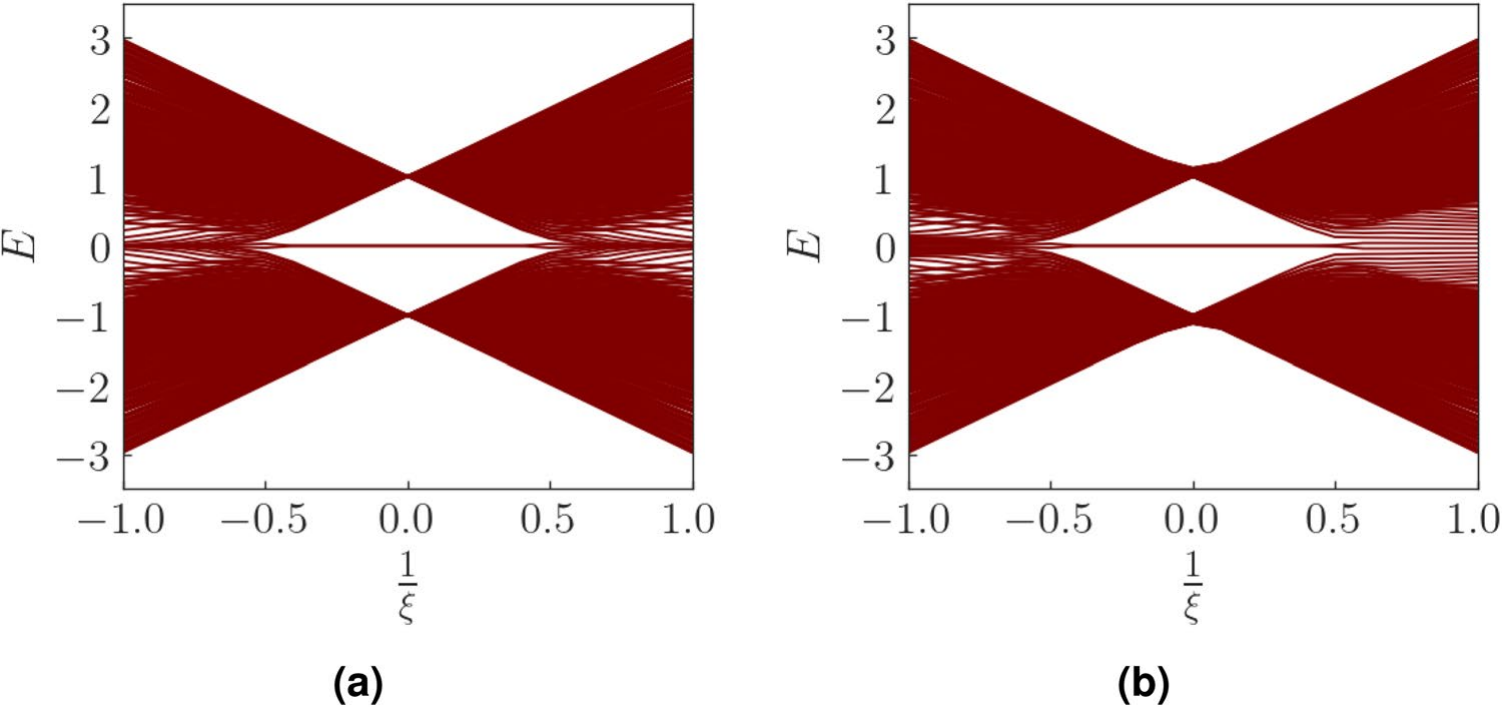
The real space eigenvalue spectra of the Hamiltonian for the rhombic supercell for (**a**) $t_2=0$ and (**b**) $t_2=0.1$. Evidently, the SOTI phase remains unaffected in the presence of the second neighbour hopping. Other parameters are kept the same as previously mentioned.

where $|w_n\rangle$ is the Wannier function corresponding to the $n^{th}$ band. Here we have taken the electronic charge *e* to be 1. *S* is the area of the 2D BZ of the honeycomb lattice which in this case is equal to $\frac{8\pi ^2}{3\sqrt{3}a_0^2}$. It can be seen that the polarization is directly related to the Berry connection of the system. Owing to the gauge dependence of the Berry connection, the polarization is defined modulo a lattice vector. We calculate $p_{\alpha =x,y}$ as a function of $\xi$ for our deformed Haldane model (Fig. 12). $p_y$ is found to be perfectly quantized at $\frac{a_0}{2}$ for $\xi >2$. For $\xi <2$ (TI phase), the value of $p_y$ is not quantized. In case of the polarization along the *x* direction, we find that it is uniformly zero both for the TI and SOTI phase. The quantized value of $p_y$ at $\frac{a_0}{2}$ indicates a discrepancy between the positions of the WCC and the lattice site. This indicates a non-trivial bulk topology. The quantization of the polarization $p_y$ is due to the presence of inversion symmetry in the system. Inversion symmetry (which is only present when $m=0$) allows an interchange of the A and B sublattices, while keeping the center of the strong bond invariant. This causes the center of charge to be localized at the center of the strong bond and results in $p_y$ to be quantized. The presence of $M_x*T$ symmetry which maps the corners (at which localization occurs) of the rhombus onto themselves, further augments the robustness of the higher order topological states.

## Analysis of the second order topological phase

It is interesting to note that while, the second neighbour hopping $t_2$ plays an important role in deciding the fate of topology in the TI phase ($\xi <2$), this is not the case for the SOTI phase ($\xi >2$).The bandstructure corresponding to the TI phase shows a distinct gap opening transition in going from $t_2=0$ to $t_2\ne 0$. The bandstructures in the SOTI phase show no such transition indicating that the Hamiltonian with $t_2\ne 0$ is adiabatically connected to the Hamiltonian with $t_2=0$ in the SOTI phase (Fig. 13). The real space eigenvalue spectra for the rhombic supercell also shows that the SOTI phase is unaffected by the presence of $t_2$ (Fig. 14). This allows us to put $t_2=0$ and hence make the system time reversal symmetric. The Hamiltonian (Eq. 2) can now be written as:

8 $$ \begin{aligned}{}&H(\vec k) = \begin{pmatrix} 0&{}G(\vec k)\\ G^*(\vec k)&{}0\\ \end{pmatrix} \end{aligned}  $$

Here

9 $$\begin{aligned}  G(\vec k)&=\Big [t_1\cos \vec {k}\cdot \vec {\delta _1} + \sum _{i=2,3}t\cos {\vec {k}\cdot \vec {\delta _i}}\Big ] - i\Big [t_1\sin \vec {k}\cdot \vec {\delta _1} + \sum _{i=2,3}t\sin {\vec {k}\cdot \vec {\delta _i}}\Big ] \end{aligned}  $$

The eigenvalues for the Hamiltonian are $\pm |G|$ and the ground state eigenvector is given by $|\Psi _g\rangle =(-e^{i\theta }, 1)/\sqrt{2}$, where $\theta =-i\text {ln}(G/|G|)$. The expression for the polarization thus becomes,

10 $$ \begin{aligned} p_\alpha ={{\bar{r}}_\alpha } =\frac{i}{S}\int _{BZ} d^{d}k \frac{i\partial \theta }{\partial k_{\alpha }} \end{aligned}  $$

Now following the argument that the topological invariant does not change unless there is a gap closing transition, we explicitly calculate polarization for a special situation $t=0$ such that $t_1$ remains greater than 2*t*. Thus,

11 $$\begin{aligned}  G(\vec k)&=t_1(\cos \vec {k}\cdot \vec {\delta _1}-{i}\sin \vec {k}\cdot \vec {\delta _1}) \end{aligned}  $$

**Figure 15:**
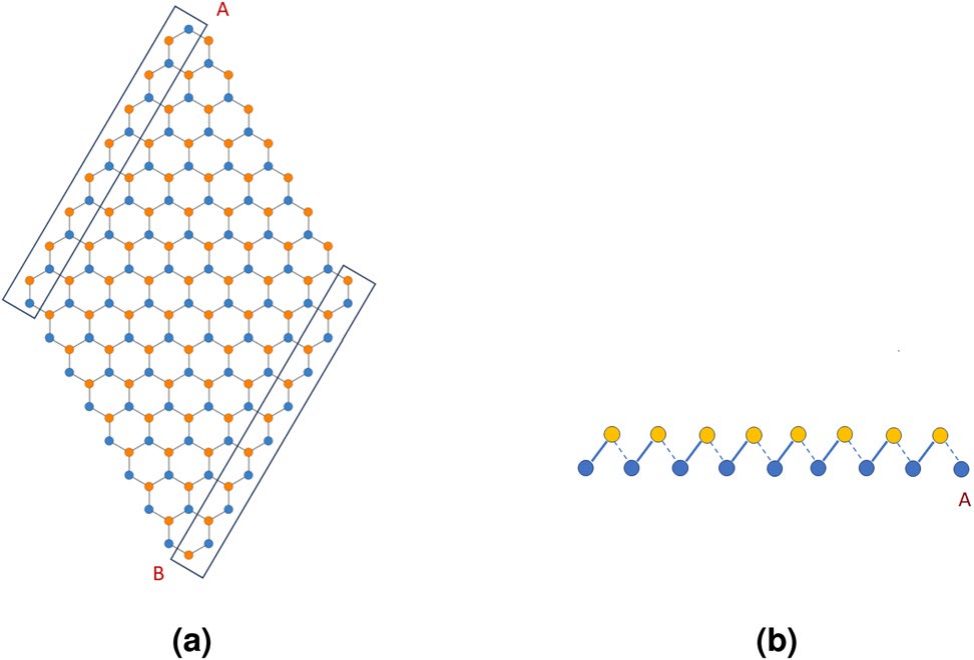
The rhombic supercell in the SOTI phase bears an analogy with the two dimensional SSH model. (**a**) Two of the edges (upper and lower) hosting an uncompensated weak bond at one end are marked. (**b**) The upper edge (marked) of the rhombus is shown. The dominant bonds with hopping amplitude $t_1$ are shown by a solid line and the weak bonds are shown by dashed lines. This edge ends at a site A, which is connected via weak bonds to the rest of the system, making it a host to the localized higher order topological state.

The ground state for this Hamiltonian is given by $|\Psi _g\rangle =(-e^{-ik_ya_0}, 1)/\sqrt{2}$. Evidently the polarization vector assumes the value ${\varvec{P}}=(p_x, p_y)=(0, \frac{a_0}{2})$. It is interesting to note that the topology of the SOTI phase bears an analogy with the topology of the Su–Schrieffer–Heeger (SSH) model. Beyond $\xi =2$, the rhombic supercell behaves like a two dimensional SSH model where the sites boxed in Fig. 15 act as a single chain and the ones parallel to these constitute sites in the other dimension. There is one subtle difference with the traditional one dimensional SSH model where a trivial to topological transition occurs at $\xi =1$. However, in this case the transition from a TI (trivial SOTI) to an SOTI phase occurs at $\xi =2$. The sites marked as *A* and *B* as shown in Fig. 15 are connected to the bulk of the system by weak bonds causing the zero energy states to localize there. Further, this second order topological phase is an example of a topological phase with zero Berry curvature. The absence (presence) of time reversal symmetry or a non-zero $t_2$ ($t_2=0$) is not crucial for the SOTI phase as evident from Fig. 13c,d, owing to the adiabatic connection of the Hamiltonians. Therefore, we assume time reversal symmetry to be prevalent in the system. The combined action of time reversal symmetry (TRS) and inversion symmetry (IS) forces the Berry curvature to be zero uniformly across the Brillouin zone^3^. IS requires that $\Omega (-\vec {k})=\Omega (\vec k)$, whereas TRS requires that $\Omega (-\vec {k})=-\Omega (\vec k)$, which results in the vanishing of the Berry curvature. However, the Berry connection is non-zero, yielding the signature of a topological phase with zero Berry curvature.

## Conclusion

In this work, we have studied a band deformed Haldane model by smoothly tuning one of the three nearest neighbour hopping amplitudes in a honeycomb lattice. This band deformation breaks the $C_3$ symmetry of the original Haldane model and causes the Dirac nodes at the **K** and **K**$'$ points in the BZ to shift and move towards an intermediate **M** point as $t_1$ (the nearest neighbour hopping amplitude along $\hat{\delta }_1$) approaches 2*t* (where *t* is the nearest neighbour hopping amplitude along $\hat{\delta }_2$ and $\hat{\delta }_3$). We show that this causes a shift in the concentration of Berry curvature in the BZ as well. We calculate Chern number and plot it as a function of $\xi$ ($=\frac{t_1}{t}$) to show that it has a value of 1 for $\xi <2$ while it becomes zero beyond that. We establish that the region $\xi >2$ is a higher order topological phase, as contrary to earlier works which reported this phase as trivial owing to the absence of edge states in the system. We further calculate bulk polarization ($p_{\alpha =x,y}$) for this model, which serves as a topological invariant. $p_y$ acquires a constant value of $\frac{a_0}{2}$ while $p_x$ remains zero for the entire SOTI phase. This quantization is a consequence of the inversion symmetry which remains intact in the system as long as the Semenoff mass is zero. Finally, we show that the SOTI phase bears an analogy with the two dimensional SSH model and establish that the presence or absence of time reversal symmetry is inconsequential to the SOTI phase making it a classic case of a topological phase with zero Berry curvature.

## Data Availability

All data that support the findings of this study are included within the article.
